# Type 1 Retinopathy of Prematurity and Its Laser Treatment of Large Preterm Infants in East China

**DOI:** 10.1371/journal.pone.0144313

**Published:** 2015-12-16

**Authors:** Haidong Shan, Yinqing Ni, Kang Xue, Jia Yu, Xin Huang

**Affiliations:** 1 Department of Ophthalmology and Visual Science, Eye and ENT Hospital of Fudan University, Shanghai 200031, China; 2 Shanghai Key Laboratory of Visual Impairment and Restoration, Fudan University, Shanghai 200031, China; 3 Department of Ophthalmology, Kashgar Prefecture Second People’s Hospital, Xinjiang 844000, China; University of Sydney, AUSTRALIA

## Abstract

**Purpose:**

To describe Type 1 retinopathy of prematurity (ROP) and its laser treatment outcomes in premature infants with birth weight > 1250 g in Eastern China.

**Methods:**

A retrospective review of 3175 ROP records was conducted at Shanghai Eye & ENT Hospital of Fudan University. The records were collected at the ROP clinic from 2006 to 2014, including their demographic and medical information such as gestational age, birth weight, supplemental oxygen therapy, systemic complications, ROP stage, location, presence of plus disease. All infants were examined by RetCam fundus camera. Those with Type 1 ROP were also examined by indirect ophthalmoscope before undergoing transpupillary laser treatment.

**Results:**

A total of 12 infants (24 eyes) with Type 1 ROP and birth weight > 1250 g were enrolled. All infants enrolled had plus disease and ROP in zone II retina. Specifically, 16 eyes (67%) had stage 2 ROP. 8 eyes (33%) had stage 3 ROP. ROP regressed in 23 eyes (96%) following laser treatment. Partial retinal detachment developed in one eye (4%). No severe involution sequelaes or laser-related complications were recorded. Mean follow-up was 30±6 weeks.

**Conclusion:**

Type 1 ROP may occur in large premature infants who have undergone supplemental oxygen therapy. This Type 1 ROP is mainly located in zone II retina. Laser treatment is a safe and effective intervention for these infants.

## Introduction

Retinopathy of prematurity (ROP), characterized by avascular retina that may potentially lead to retinal detachment and blindness, is mostly seen in small preterm infants [1]. Screening examination is recommended in those with birth weight < 1500g in developed countries [2, 3]. However, severe ROP is occasionally reported in large preterm infants in middle-income countries such as China, where excessive oxygen therapy may contribute to its occurrence [4–7]. Thus, Chinese authorities recommend ROP screening in infants with a birth weight of < 2000g [8].

Studies have demonstrated that laser photocoagulation can significantly reduce unfavorable outcomes of severe ROP in infants with low birth weight (< 1250g) [9, 10]. However, data are limited about severe ROP in large preterm infants and their laser treatment outcomes [11–14]. It was reported that stage 3 ROP occurred in only 1–3% of infants with birth weight ≥ 1250 g [13, 15]. It is suspected that those infants may differ from low birth weight infants in response to laser treatment due to their retinal vascularization, high oxygen exposure and other systemic complications [11, 14]. In this study, we reviewed our ROP clinic records between the period 2006 to 2014 and described Type 1 ROP characteristics in a series of large premature infants. Their risk factors, ROP involution sequelaes and laser treatment outcomes were also discussed.

## Patients and Methods

### Data review and enrollment

This study was approved by the Ethics Review Board of Shanghai Eye & ENT Hospital of Fudan University, East China. A retrospective review of ROP records from 2006 to 2014 was conducted. The records were collected at our ROP clinic, including infant’s demographic and medical information such as name, sex, date of birth, gestational age, birth weight, systemic complications, history of oxygen supplementation, ROP location, stage, presence of plus disease, laser treatment and follow-up examinations. Enrollment criteria included those with birth weight > 1250g, diagnosed with Type 1 ROP, underwent laser treatment and were followed up > 12 weeks.

### Screening examination

Written informed consents were obtained from parents before screening examinations. All infants were examined using a RetCam fundus camera (Clarity Medical Systems, Pleasanton, CA) as previously described[16]. In brief, the pupil was dilated and topical anesthesia was performed. The examiner gently used a lid speculum to open the eye and added a drop of transparent gel on the cornea. The tip of the lens handle was carefully placed on the cornea. The retina was examined on live mode of the camera and images were taken as necessary. ROP was documented according to the International Committee for the Classification of Retinopathy of Prematurity[1]. Type 1 ROP is defined as: (1) any ROP with plus disease in zone I; (2) stage 3 ROP in zone I; (3) stage 2 or 3 ROP with plus disease in zone II[9].

### Laser treatment

Transpupillary laser treatments were performed within 24 hours of Type 1 ROP diagnosis. The pupil was dilated and topical anesthesia was performed. The experts gently open the eye with a lid speculum and re-examined the fundus using a 28D lens and an indirect ophthalmoscope, integrated with 532 nm laser accessories (Purepoint, Alcon, US). The photocoagulation was performed on a nearly confluent pattern in the avascular retina. Repeat mode was used with 0.2s exposure time and 0.2s interval time. Power was set to achieve white-grey burns, generally between 200 to 350 mW. Topical antibiotic eye drops were used during and after the procedure. Any ocular or systemic complications related to photocoagulation were recorded.

### Follow-up examination

Following laser treatment, infants were examined by RetCam weekly until ROP regression appeared. They were then imaged every 2–4 weeks until no active ROP existed. If ROP recurred post-treatment, additional photocoagulation or vitrectomy was applied when necessary.

### Statistic analysis

All data were analyzed using MS Excel and Prism. Fisher’s exact test was used to compare the difference of Type 1 ROP location, staging and regression rate following laser treatment between our large preterm infants and those with low birth weight in the ET-ROP study. Data were presented as mean ± SD.

## Results

**Fig 1:**
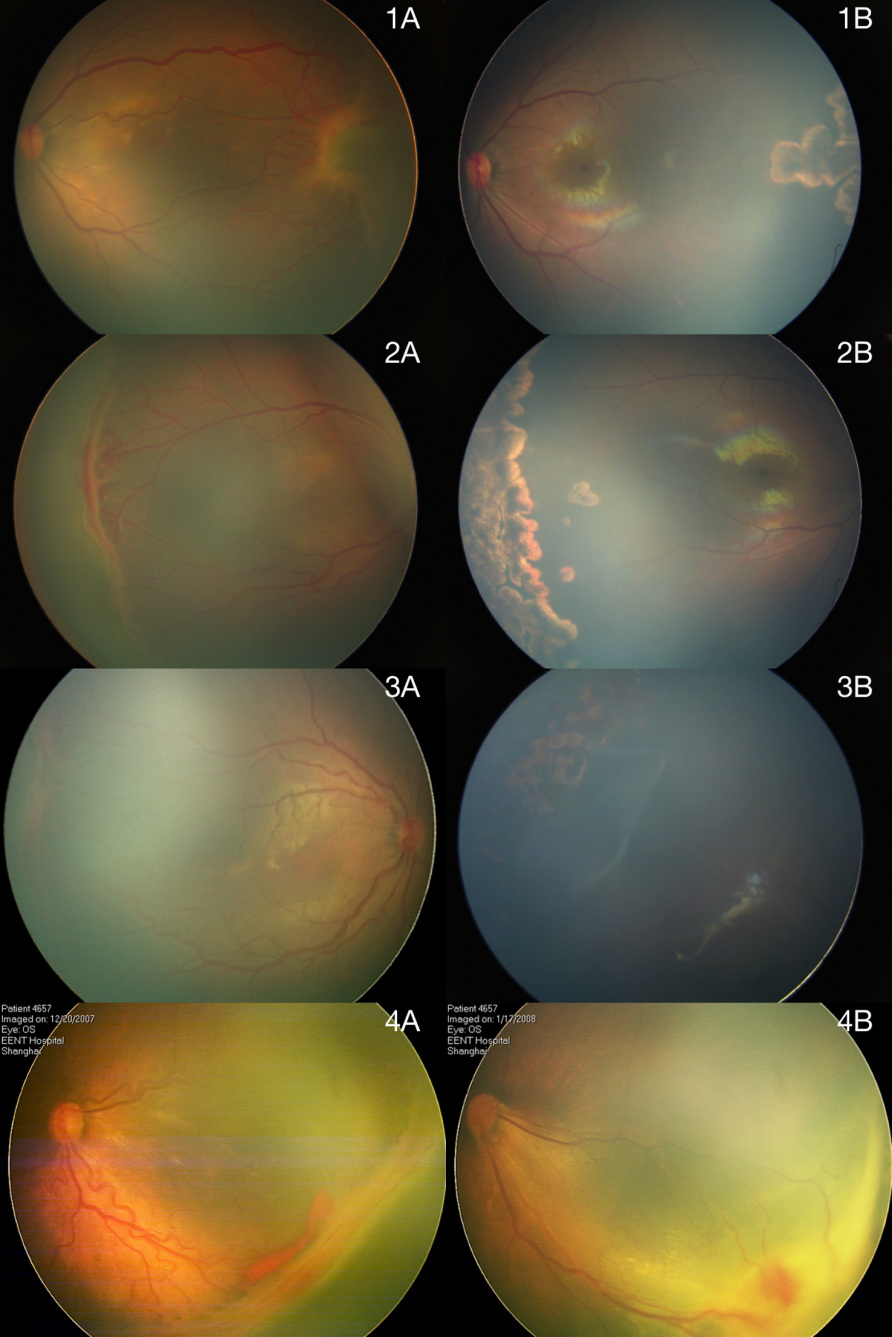
Fundus images of Type 1 ROP and its laser treatment outcomes in large preterm infants. 1A-4A: Brush-like retinal neovascularization was observed on the posterior margin of avascular peripheral retina, located in zone II retina. All eyes had increased venous dilation and arteriolar tortuosity of the posterior retinal vessels (plus disease). 1B-3B: ROP regressed following laser treatment, with absence of peripheral retinal vascularization, retinal pigmentary changes and vitreoretinal interface changes. 4B: Temporal retinal detachment developed at 4 weeks after laser treatment, associated with macular heterotopia, straightening of blood vessels in temporal arcade and dragging of retina over optic disc.

A total of 12 infants (24 eyes) were enrolled from 3175 records of our ROP clinic between the period from 2006 to 2014. All were diagnosed as Type 1 ROP and underwent laser treatment. Their mean birth weight was 1450±155g (1265–1800g). The mean gestational age was 30±2 weeks (28–34 weeks). Of all, 7 were male (58%) and 5 were female (42%). Type 1 ROP was located in zone II retina of all infants (Fig 1). Specifically, 16 eyes (67%) had stage 2 ROP. 8 eyes (33%) had stage 3 ROP. Plus disease was present in all eyes. Their demographic and ROP information are listed in Table 1. Their risk factors were analyzed in Table 2.

**Table 1:** The infants’ demographic and ROP information in the study.

No.	Sex	Gestational age (week)	Birth weight (g)	ROP location (zone)	ROP stage	Plus disease	Age at treatment (week)	Postmenstrual age at treatment (week)	Laser counts	Outcome	Follow-up (week)
**1**	Male	29	1420	OU: II	OU: 3	(+)	6	35	2495	OD: Regressed. OS: partial retinal detachment	20
**2**	Male	30	1265	OU: II	OU: 2	(+)	10	40	1457	OU: Regressed	25
**3**	Female	29	1365	OU: II	OU: 3	(+)	8	37	2158	OU: Regressed	28
**4**	Female	30	1450	OU: II	OU: 2	(+)	3	33	1405	OU: Regressed	37
**5**	Female	30	1350	OU: II	OU: 2	(+)	3	33	1664	OU: Regressed	37
**6**	Male	28	1285	OU: II	OU: 2	(+)	6	34	2515	OU: Regressed	30
**7**	Male	29	1300	OU: II	OU: 2	(+)	9	38	1207	OU: Regressed	32
**8**	Male	32	1400	OU: II	OU: 2	(+)	6	38	1314	OU: Regressed	33
**9**	Female	33	1600	OU: II	OU: 2	(+)	5	38	1105	OU: Regressed	31
**10**	Female	31	1550	OU: II	OU: 2	(+)	5	36	1050	OU: Regressed	42
**11**	Male	30	1620	OU: II	OU: 3	(+)	5	35	1054	OU: Regressed	25
**12**	Male	34	1800	OU: II	OU: 3	(+)	6	40	1205	OU: Regressed	24

ROP, retinopathy of prematurity; (+), presence of plus disease.

**Table 2:** Risk factors of Type 1 ROP in the infants of the study.

Risk factors	Number of infants (%)
**Mask oxygen supplement**	11 (92)
**Apnea**	10 (83)
**Mechanical oxygen supplementation**	9 (75)
**Pneumonia**	9 (75)
**Congenital heart disease**	5 (42)
**Neonatal respiratory distress syndrome**	3 (25)
**Septicemia**	2 (17)

All eyes underwent laser treatment at mean 6±2 weeks after birth (3–10 weeks). The mean postmenstrual age was 37±2 weeks (33–40 weeks). The infants received mean 1552±518 laser spots (1050–2515). ROP regressed spontaneously in 23 eyes (96%, Fig 1). Details of involutional sequelae are listed in Table 3. Partial retinal detachment involving macula, also known as stage 4b ROP, was found in 1 eye by RetCam and B-Ultrasonic scan at 4 weeks after laser treatment (Fig 1). No severe ocular or systemic complications were identified. Mean follow-up were 30±6 weeks (20–42 weeks).

**Table 3:** Sequelae of ROP regression following laser treatment in the study.

Changes	Number of eyes (%)
**Failure of peripheral retinal vascularization**	23 (100)
**Abnormal branching of retinal vessels**	17 (74)
**Retinal pigmentary changes**	10 (43)
**Vascular arcades with circumferential interconnection**	7 (30)
**Vitreoretinal interface changes**	4 (17)

## Discussion

Our study described Type 1 ROP and its laser treatment outcomes in a series of large preterm infants in Eastern China. All had birth weight > 1250 g and were diagnosed with stage 2 or 3 ROP in zone II retina with plus disease. Following laser treatment, ROP generally regressed with minor involution sequelaes.

**Fig 2:**
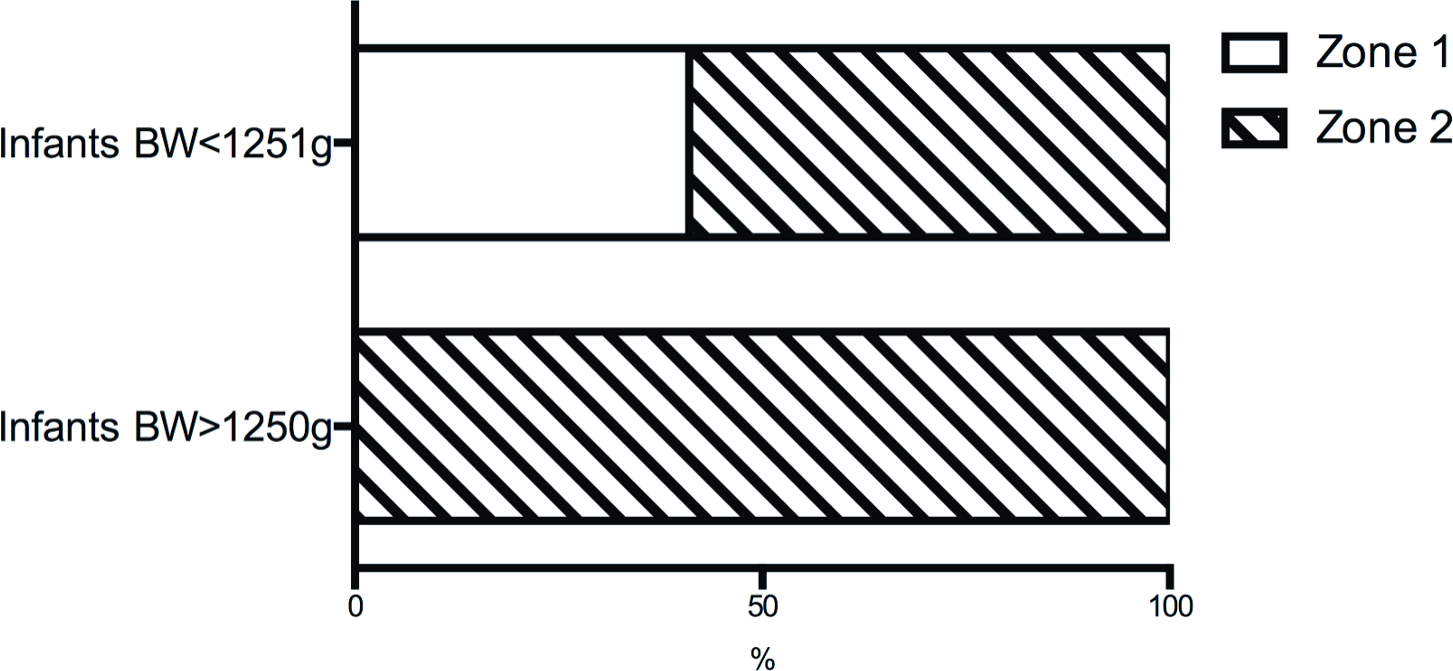
Comparison of Type 1 ROP location between large and small preterm infants. Type 1 ROP was in zone II retina of all eyes in the study, while 41% of them were in zone I retina of the small preterm infants. BW: birth weight. Data source of infants BW < 1251 g: the ET-ROP study [10].

**Fig 3:**
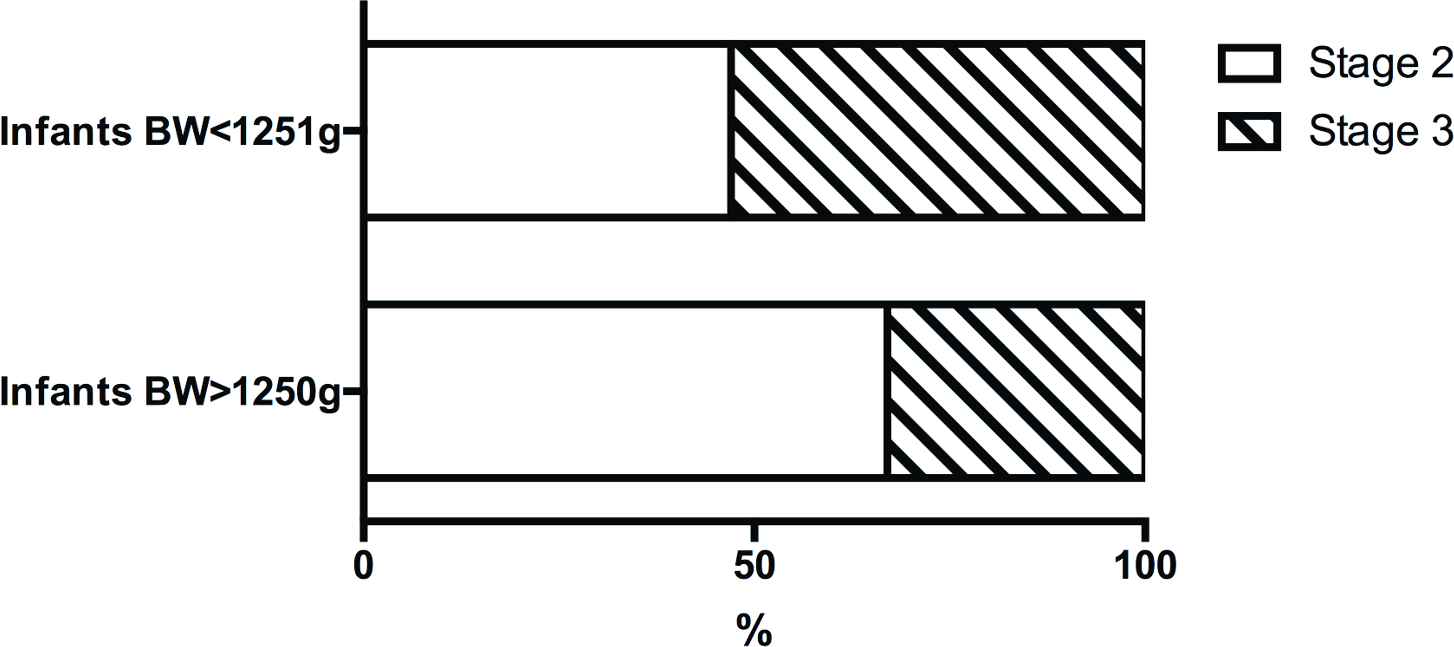
Comparison of Type 1 ROP stage between large and small preterm infants. The percentage of stage 3 ROP was significantly lower in large preterm infants than that of small preterm infants (33% vs. 53%, p = 0.007). BW: birth weight. Data source of infants BW < 1251 g: the ET-ROP study [10].

We compared our results with the outcomes of the ET-ROP study to determine any difference of Type 1 ROP characteristics between low and large birth weight infants in our cohort of ROP infants. In the ET-ROP study, infants with birth weight <1251 g and high-risk prethreshold ROP were randomized to treatment or observation[9]. Of all 361 treated eyes, 41% had ROP in zone I retina and 59% was in zone II. Stage 3 ROP was found in 53% of all treated eyes. In contrast, Type 1 ROP in our study was located in zone II retina of all eyes (Fig 2). No ROP identified was in zone I. Meanwhile, the percentage of stage 3 ROP was significantly lower in the large preterm infants of our study than that seen in the small preterm infants of the ET-ROP study (33% vs. 53%, p = 0.007, Fig 3).

The difference of ROP location and stage between large and small preterm infants may well be due to the disparities in their vascular maturity and development after birth. According to the results, our large preterm infants were almost 5 weeks older than those in the ET-ROP study at gestational age (30±2 vs. 25±1 weeks). Hughes et al made a comprehensive study of retinal vascularization in human fetal retina [17]. They found immature vascular cords are formed from the optic disc between 14–15 weeks of gestation (WG). The inner vascular plexus reaches its outer limits by 32 WG. In contrast, formation of the outer plexus begins in the perifoveal region between 25–26 WG and subsequently spread to the peripheral retina by about 40 WG. By CD34 immunostaining, they concluded that vasculogenesis contributes to the formation of the inner plexus, whilst angiogenesis is responsible for the vascular spread and density in human retina. Similar results were also published by Stone and Chan-Ling et al [18, 19]. Therefore, our large preterm infants could have gained additional intrauterine time for retinal vascularization that could contribute to their ROP location and staging difference from the infants with birth weight < 1251 g in the ET-ROP study.

However, our results indicate that those large preterm infants are still susceptible to severe ROP, particularly when they have risk factors such as supplemental oxygen therapy. In the study, 92% of infants had mask oxygen supplementation and 75% had mechanical oxygen therapy. Apnea occurred in 83% of infants enrolled in the study. In small preterm infants, however, only 24% had systemic complications such as apnea and reincubation [9]. Other studies also suggest that large preterm infants with severe ROP seem to suffer systemic complications including prenatal blood loss [12, 14], sepsis[13, 20], anaemia and hypoxic-ischemic encephalopathy [21]. Thus, these multiple risk factors and unmonitored supplemental oxygen for prolonged duration may contribute to severe ROP occurrence of large preterm infants in developing countries such as China [22].

**Fig 4:**
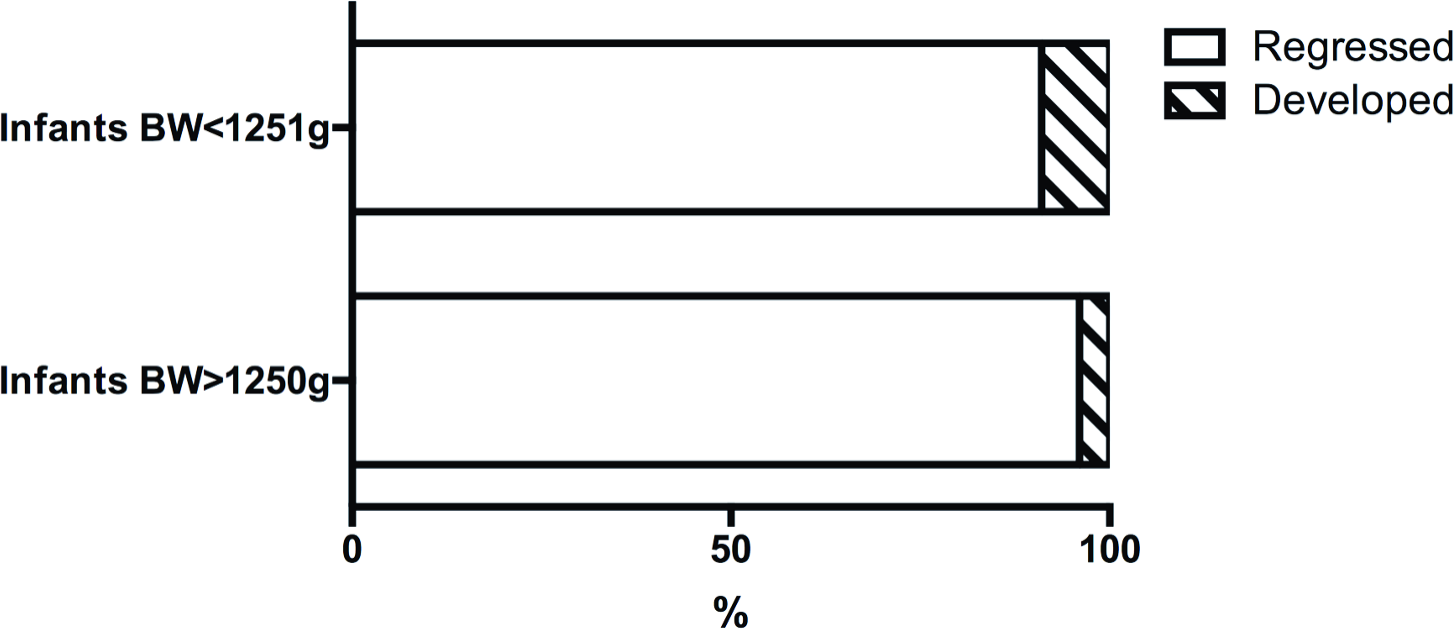
Comparison of Type 1 ROP evolution between large and small preterm infants following laser treatment. ROP regressed in similar percentage of eyes in large and small preterm infants (96% vs. 91%, p = 0.25). BW: birth weight. Data source of infants BW < 1251 g: the ET-ROP study [10].

Fortunately, our study indicates laser treatment can achieve similar favorable outcomes in large preterm infants when compared with small preterm infants. Actually, the ROP regression rate following laser treatment in our study is slightly higher than that of the ET-ROP study, although no statistic significance was found (96% vs. 91%, p = 0.25, Fig 4). This could be mainly caused by the characteristics of Type 1 ROP in large preterm infants, as all Type 1 ROP were found in zone II retina in this study. Laser treatment is believed to be less effective for ROP in zone I retina, particularly aggressive posterior retinopathy of prematurity (AP-ROP) [1]. In fact, the only eye in our study which developed retinal detachment was later confirmed as AP-ROP. Sanghi et al described a series of AP-ROP infants with birth weight up to 2000 g [4]. Almost one-third of those infants developed retinal detachment despite confluent laser photocoagulation. In another report, AP-ROP occurred in 15 infants with birth weight >1500 g [11]. Nearly 25% of these AP-ROP cases resulted in unfavorable outcomes. Flynn and Chan-Ling believe that zone I ROP is correlated with vasculogenesis so it is therefore insensitive to laser or cryotherapy [23]. These reports, together with our study, draw attention that AP-ROP can occur in large preterm infants. Additional treatments, such as intravitreal injection of anti-VEGF drugs or early vitreous surgery, need to be addressed for AP-ROP in large preterm infants as well as in small preterm infants [24–26]. This is particularly important when infants do not respond well and show signs of disease progression following laser treatment.

This study also reveals some difference of chronological and postmenstrual age between large and small preterm infants when laser treatment was performed. Our large preterm infants underwent laser treatment at 6±2 weeks of chronological age, which is about 4 weeks earlier than those in the ET-ROP study (10±2 weeks). Meanwhile, large preterm infants were 2 weeks older than those with BW < 1251 g in the ET-ROP study in terms of the postmenstrual age when laser was applied (37±3 vs. 35±2 weeks). Similar results were also reported by Chen et al [22]. These data suggest large preterm infants seem to have severe ROP sooner after birth than small preterm infants, which should be considered when doctors decide the date of first fundus examination in large preterm infants. This may also help the authorities of developing countries design ROP screening guidelines, such as in China.

Overall, our study demonstrates Type 1 ROP may occur in infants with birth weight >1250 g. Laser photocoagulation is a safe and effective intervention for those infants. Additional interventions should be considered for those who do not respond well and show signs of progression following laser treatment.
